# High-Dose Oral Amiodarone for Cardioversion of Atrial Fibrillation: A Case Report

**DOI:** 10.7759/cureus.41766

**Published:** 2023-07-12

**Authors:** Giovanni Reynaldo, Rachmat Hamonangan, Pingkan Naomi, Nicolas Daniel Widjanarko

**Affiliations:** 1 Internal Medicine, St. Carolus Hospital, Jakarta, IDN; 2 General Practice, Atma Jaya Catholic University, Jakarta, IDN

**Keywords:** high-dose amiodarone, arrhythmia, anti-arrhythmic drugs, cardioversion, atrial fibrillation

## Abstract

With the rising prevalence of atrial fibrillation (AF), it has become a global health problem with increasing complications and high medical costs. Here, we describe the case of a 52-year-old woman with chest discomfort and frequent palpitations for the last few months. A careful clinical and instrumental examination showed that the patient had AF. Sinus rhythm was restored by cardioversion using high-dose oral amiodarone therapy. Although this medication can be an alternative with several advantages over electrical cardioversion in the future, further studies are needed to establish its efficacy and safety profile.

## Introduction

Globally, the average life expectancy is rising, which allows people to live longer with chronic diseases [1]. The most frequent cardiac arrhythmia found in clinical practice is atrial fibrillation (AF) [2]. The frequency and incidence of AF are increasing, making it a global health issue. According to estimates, by 2050, there will be 17.9 million individuals living with this illness in Europe and 6-12 million in the United States [1,2]. For the past 10 years, the prevalence of AF among those over the age of 70 or 80 has more than doubled, from 4.6% to 8.2%. For every year group, men had a greater frequency of developing AF than women. In the Asia-Pacific region, AF epidemiological studies have reported a prevalence ranging from 0.49% to 5.4% [1]. Although the prevalence has significantly increased, it remains lower than in many Western countries [3]. Unfortunately, the burden of AF is predicted to be greater in this region compared to North America and Europe due to the region’s aging population. To execute effective screening and targeting techniques for preventative and therapeutic treatment, precise and cautious procedures are needed. This report details a case of unstable AF that was successfully cardioverted using a high dosage of oral amiodarone.

## Case presentation

A 52-year-old female patient arrived at the hospital’s outpatient department with complaints of pain, pressure, fullness in the chest, and frequent palpitations for the last few months. The patient added that since a week prior, her left arm and leg had frequently felt numb. Diabetes, hypertension, or any other ailments were unknown in the previous examination. No smoking history was reported by the patient.

**Figure 1:**
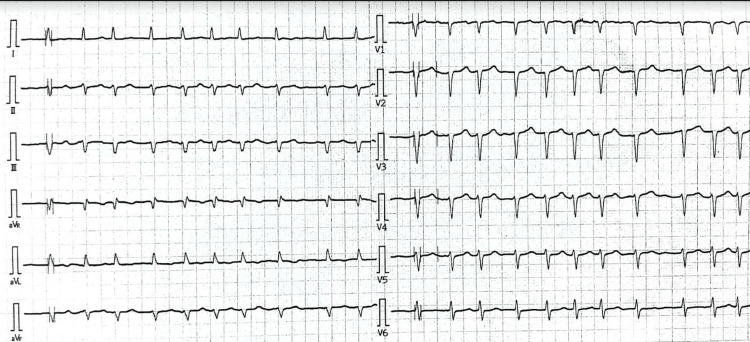
Electrocardiography showing an irregular pulse with a diagnosis of atrial fibrillation.

The physical examination revealed irregular heart sounds, with hypesthesia in the left limb. The results of additional examinations obtained from electrocardiography showed AF (Figure 1) with a heart rate of 120-150 beats/minute, blood pressure of 150/90 mmHg, and SpO_2_ of 92%. Echocardiography showed an ejection fraction of 57% and left atrial enlargement. Magnetic resonance imaging (MRI) of the head showed an infarction in the right parieto-occipital region. Blood examinations were performed, and the results were within the normal range except for an increase in low-density lipoprotein levels. The complete results of blood examinations can be seen in Table 1.

**Table 1:** Results of the patient’s blood examinations.

Parameter	Results	Normal range
Hemoglobin	13.8 g/dL	13.5–16 g/dL
Low-density lipoprotein	157 mg/dL	<100 mg/dL
Triglycerides	101 mg/dL	<150 mg/dL
Creatinine	0.91 mg/dL	0.6–1.2 mg/dL
Potassium ion	4.4 mmol/L	3.7–5.2 mmol/L
Calcium ion	1.14 mmol/L	2.2–2.6 mmol/L
Magnesium ion	2.10 mg/dL	1.8–2.5 mg/dL
Free thyroxine	1.41 ng/dL	0.7–1.55 ng/dL
Thyroid-stimulating hormone	3.52 µIU/mL	0.27–4.2 µIU/mL

The patient was given therapy with an initial dose of amiodarone 2,000 mg, followed by titrated maintenance doses of 4 × 200 mg, 3 × 200 mg, and 2 × 200 mg; atorvastatin 40 mg; rivaroxaban 20 mg; and imidapril HCl 10 mg. The results of follow-up examinations at the clinic showed a conversion to sinus rhythm six hours post-cardioversion.

## Discussion

As the most prevalent persistent arrhythmia, AF is characterized by erratic, fast, and uncoordinated atrial activity. While atrioventricular (AV) conduction remains intact, the electrocardiogram of AF is distinguished by the substitution of normal P-waves with quick oscillations that vary in size, shape, and timing. These oscillations are also accompanied by an irregular, fast ventricular response. Over the next 40 years, it is predicted to impact more than twice as many people. Moreover, it is linked to early death and significant serious adverse cardiovascular events such as heart failure, severe stroke, and myocardial infarction [4]. Based on chronology, underlying etiology, and associations with valvular heart disease, several classifications have been proposed. Due to numerous illness processes, the pathophysiology of the disease is thought to be complicated. The precise mechanism remains unknown, but it is believed to involve a complex interaction between structural and functional abnormalities (referred to as “drivers”) that promote and maintain re-entry electrical impulses, “triggers” initiating focal ectopic activity in the atria, and the presence of an abnormal substrate that sustains arrhythmia. Around the orifice of pulmonary veins, which are said to play a crucial role in the onset and maintenance of AF, sustained types of micro re-entry as drivers have also been described [5]. The role these drivers play in maintaining tachycardia may explain the success of pulmonary vein isolation procedures in eliminating more chronic or persistent forms of AF [6].

Patients are often not aware of the risk factors and the occurrence of AF, which makes early detection challenging. If a patient has AF, other potentially fatal illnesses including heart failure [7], myocardial infarction, stroke, or hemodynamic instability may develop with little or no warning [8]. Advanced age, an elevated body mass index, a history of diabetes mellitus and hypertension, smoking, and prior heart diseases are some of the main risk factors that are very likely to cause AF [9]. To pinpoint risk factors, comorbidities, and clinical findings of AF, detailed histories and physical examinations are required. In our case, the patient had minor symptoms and no prior concomitant conditions. MRI revealed ischemic infarction in the right parieto-occipital area, and the patient also reported experiencing numbness in her left limbs. The patient was evaluated using the CHA2DS2-VASc score to more accurately stratify the risk of thromboembolic events and the usage of anticoagulant treatment. The patient was further identified as high risk due to the score of +3 [10]. Anticoagulant therapy was considered and given to the patient to prevent future risk of thromboembolic events.

For individuals with AF, anti-arrhythmic medications continue to be the cornerstone of treatment. The traditional view of the optimum pharmacotherapy for AF is an anti-arrhythmic medication that can preserve sinus rhythm and restore it with a few, manageable side effects. Drug therapy and ablation therapy are the two methods of treatment for sinus rhythm maintenance. Due to its invasive nature, lack of availability, high cost, and modest but real risk of serious and life-threatening consequences, ablation treatment is often catheter-based and rarely employed as the first-line option [11]. Antiarrhythmic medications have typically been categorized according to the Vaughan-Williams (VW) classification system based on their effects on certain ion channels and receptors [12]. In stable patients, either pharmacological cardioversion or electrical cardioversion can be attempted. Although less effective, pharmacological cardioversion does not require sedation [13]. We opted for pharmaceutical cardioversion in this case as the patient’s physical state was stable. Initiating pharmacological cardioversion within seven days of the start of an AF episode is also beneficial [14].

Amiodarone is a well-known and effective antiarrhythmic medication used to treat AF and ventricular arrhythmias. This medicine reduces the recurrence of life-threatening arrhythmias and causes a modest decrease in sudden mortality in high-risk individuals. Amiodarone is a class III drug according to the VW classification because it prolongs the QT interval. Additionally, it slows intracardiac conduction (through sodium channel blockage), lowers heart rate and atrioventricular nodal conduction through the inhibition of calcium channels and beta-receptors, and prolongs refractoriness by blocking the potassium and sodium channels [15]. Electrical cardioversion is widely regarded as the gold standard procedure to restore sinus rhythm in recent-onset AF, with success rates of more than 80% [16]. However, one drawback of this approach is that it necessitates general anesthesia, which makes it difficult to perform surgeries in emergency settings [16]. Clinical trials have explored various antiarrhythmic medications, both orally and intravenously, with varied degrees of efficacy. Given its ease and possible cost savings, oral administration may be the chosen course of therapy [17]. Following the 2019 American Heart Association (AHA) guidelines for the diagnosis and treatment of AF, amiodarone can be used to restore sinus rhythm when administered orally with an initial dosage of 1.2-1.8 g per day in divided doses until 10 g total, followed by 200-400 mg per day maintenance or 30 mg/kg as a single dose. In comparison, according to the 2020 European Society of Cardiology (ESC) guidelines for the diagnosis and therapy of AF, amiodarone can also be taken intravenously with an initial dosage of 5-7 mg/kg over one to two hours and a subsequent dosage of 50 mg/hour for 24 hours of maintenance (a maximum of 1.2-1.8 g for 24 hours). No differences in intravenous dose recommendations have been reported in the newest 2020 ESC guidelines and 2019 AHA guidelines. All AF patients should take long-term rhythm management medications as increasing and advanced age, female sex, renal and/or hepatic impaired function, and previously diagnosed coronary artery have been linked to a greater risk of recurrent arrhythmia [18]. Amiodarone is advised as a long-term antiarrhythmic medication by the ESC for all AF patients, including those with heart failure with reduced ejection fraction (Grade 1A recommendation). Amiodarone is administered three times a day for four weeks at a dose of 200 mg, followed by 200 mg daily [18, 19]. Furthermore, in 2011, per the American College of Cardiology/AHA/Heart Rhythm Society focused updates incorporated guidelines for the management of patients with atrial fibrillation, 800 mg amiodarone once daily for one week was recommended [14].

Doses of oral high-dose amiodarone remain debatable, but patients treated with amiodarone at a dose of 30 mg/kg body weight showed a 98% success rate in returning to normal heart rate [20]. In our case, the patient successfully resumed her normal heart rate after three days of follow-up after receiving a loading dosage of amiodarone 2,000 mg (the patient’s body weight was 65 kg). High doses of oral amiodarone have been associated with various adverse effects, ranging from minor adverse effects such as nausea, vomiting, and diarrhea to more serious ones such as ventricular tachycardia, symptomatic bradycardia, or transient ischemic attack/stroke [21]. In our case, the patient reported no side effects. We hypothesize that such symptoms appear along with the increasing doses.

The advantage of high-dose oral amiodarone has been reported in a group of patients with chronic AF, whereas amiodarone administered as pre-treatment before electrical cardioversion enhances the likelihood of successful cardioversion [22]. There is no established recommendation for the use of high-dosage oral amiodarone as a therapy for AF due to the paucity of available data. To normalize heart rate in new-onset AF, high-dose amiodarone can be utilized instead of electrical cardioversion. Therefore, we suggest further research to determine its appropriate use in light of its effectiveness and tolerability.

## Conclusions

This case report supports the current literature suggesting that high-dose oral amiodarone can be used as an alternative to electrical cardioversion for the treatment of new-onset AF. Close monitoring of several systemic side effects such as hypotension, bradycardia, QT prolongation, gastrointestinal upset, and constipation should be performed alongside the increasing doses. Further, a novel consensus should be made to establish the safety profile of amiodarone usage worldwide.
